# An evidence map of systematic reviews on models of outpatient care for patients with chronic heart diseases

**DOI:** 10.1186/s13643-023-02227-z

**Published:** 2023-05-06

**Authors:** Madlen Hoerold, Heike Heytens, Carla Maria Debbeler, Saskia Ehrentreich, Thomas Rauwolf, Alexander Schmeißer, Marc Gottschalk, Eva Maria Bitzer, Ruediger C. Braun-Dullaeus, Christian J. Apfelbacher

**Affiliations:** 1 Institute of Social Medicine and Health Systems Research, Medical Faculty, Otto von Guericke University Magdeburg, Leipziger Str. 44, Magdeburg, Sachsen-Anhalt 39120 Germany; 2 Department of Angiology and Cardiology, Otto-von-Guericke University of Magdeburg, Leipziger Str. 44, Magdeburg, Sachsen-Anhalt 39120 Germany; 3 Department of Public Health and Health Education, University of Education Freiburg, Kunzenweg 21, Freiburg, Baden-Würtemberg 79117 Germany

**Keywords:** Heart failure, Coronary heart disease, Atrial fibrillation, Outpatient, Systematic review

## Abstract

**Background:**

Chronic heart disease affects millions of people worldwide and the prevalence is increasing. By now, there is an extensive literature on outpatient care of people with chronic heart disease. We aimed to systematically identify and map models of outpatient care for people with chronic heart disease in terms of the interventions included and the outcomes measured and reported to determine areas in need of further research.

**Methods:**

We created an evidence map of published systematic reviews. PubMed, Cochrane Library (Wiley), Web of Science, and Scopus were searched to identify all relevant articles from January 2000 to June 2021 published in English or German language. From each included systematic review, we abstracted search dates, number and type of included studies, objectives, populations, interventions, and outcomes. Models of care were categorised into six approaches: cardiac rehabilitation, chronic disease management, home-based care, outpatient clinic, telemedicine, and transitional care. Intervention categories were developed inductively. Outcomes were mapped onto the taxonomy developed by the COMET initiative.

**Results:**

The systematic literature search identified 8043 potentially relevant publications on models of outpatient care for patients with chronic heart diseases. Finally, 47 systematic reviews met the inclusion criteria, covering 1206 primary studies (including double counting). We identified six different models of care and described which interventions were used and what outcomes were included to measure their effectiveness. Education-related and telemedicine interventions were described in more than 50% of the models of outpatient care. The most frequently used outcome domains were death and life impact.

**Conclusion:**

Evidence on outpatient care for people with chronic heart diseases is broad. However, comparability is limited due to differences in interventions and outcome measures. Outpatient care for people with coronary heart disease and atrial fibrillation is a less well-studied area compared to heart failure. Our evidence mapping demonstrates the need for a core outcome set and further studies to examine the effects of models of outpatient care or different interventions with adjusted outcome parameters.

**Systematic review registration:**

PROSPERO (CRD42020166330).

**Supplementary Information:**

The online version contains supplementary material available at 10.1186/s13643-023-02227-z.

## Background

Chronic heart diseases (we consider heart failure, coronary heart disease, and atrial fibrillation) are complex clinical conditions associated with various symptoms and comorbidities such as dyspnea, fatigue, peripheral edema, and depression [1, 2]. Coronary heart disease (CHD), heart valve disease, arrhythmias, familial cardiomyopathy, toxin‐induced cardiomyopathy, and hypertension are all linked to heart failure (HF) [1]. Reported estimates of heart failure incidence in European countries and the USA ranges widely from100/100,000 person-years, in French to 4300/100,000 person-years in a US study and strongly depends, on the population studied and the diagnostic criteria used [3]. Incidence increases with age and with ageing populations this means that prevalence is also on the increase [3]. At least 26 million people are affected worldwide [4]. Coronary heart disease is the leading cause of death in both developed and developing countries. Considering current lifestyles, the incidence of CHD will continue to rise [5]. Atrial fibrillation (AF) is the most prevalent arrhythmia managed in clinical practice and one of the leading causes of HF. The 2019 Global Burden of Disease Study showed that there were about 59.7 million individuals with atrial fibrillation/flutter worldwide [6]. The majority of patients with heart failure exhibit multi‐morbidity and the number of patients with three or more chronic comorbidities increased [7]. Comorbidity is associated with increased severity of HF symptoms and corresponds to a poor quality of life and a worse prognosis [8].

Patients with chronic HF increasingly receive outpatient care. However, they are frequently hospitalised for acute decompensated as well as non‐cardiovascular causes. Assessment of prognosis of heart failure is particularly challenging. The clinical course depends on the underlying pathomechanisms and varies depending on the severity of the disease. Outcomes are difficult to predict in individual patients. Even late in heart failure, patients still have periods of “good days” and apparent stability, which can lead them and their care providers into postponing vital decisions [9]. Therapeutic interventions in each disease stage aim to modify risk factors, treat risk and structural heart disease to prevent HF, and reduce symptoms, morbidity, and mortality [10]. Holistic management approaches must foster the implementation of multidisciplinary approaches to address major contributors to the persisting burden of HF including multimorbidity, ageing, and social determinants of health [11]. Heart failure treatment constitutes challenges related to both self-care and emotional burden. Many patients are struggling emotionally due to a lack of information and education, inadequate care coordination and troublesome medication and self-monitoring of symptoms. Doing so affects their self-care ability and their well-being as well as their quality of life [12].

There is a large body of literature on the care provided to people with chronic heart diseases. However, previous systematic reviews (SRs) have mainly focused on the effects of specific health services.

Model of care is an overarching design for the provision of a particular type of health care service that is shaped by a theoretical basis, evidence-based practice, and defined standards. It consists of defined core elements and principles and has a framework that provides the structure for the implementation and subsequent evaluation of care. Clearly defined models of care help to ensure that all health professionals are working towards common goals and, most importantly, to evaluate service on agreed outcome measures [13].

This evidence map [14, 15] thus has an important purpose. We aimed to identify models of outpatient care (MoC) associated with chronic heart diseases in published systematic reviews (SRs), and to map which interventions built these models of care as well as the outcomes measured and reported. This will help to identify gaps and future research needs. A broad systematic review of primary studies was not feasible within a reasonable timescale. Hence, we decided to conduct an overview (evidence map) of systematic reviews. This approach [16–18] is increasingly used in research areas where the literature has already been summarised in several systematic reviews.

### Objectives

1. To identify any specific models of outpatient care for patients with chronic heart diseases, in systematic reviews, published in the English or German language.
2. Create a comprehensive overview (evidence map) of identified interventions and outcomes.

## Methods

### Protocol and registration

**Fig. 1:**
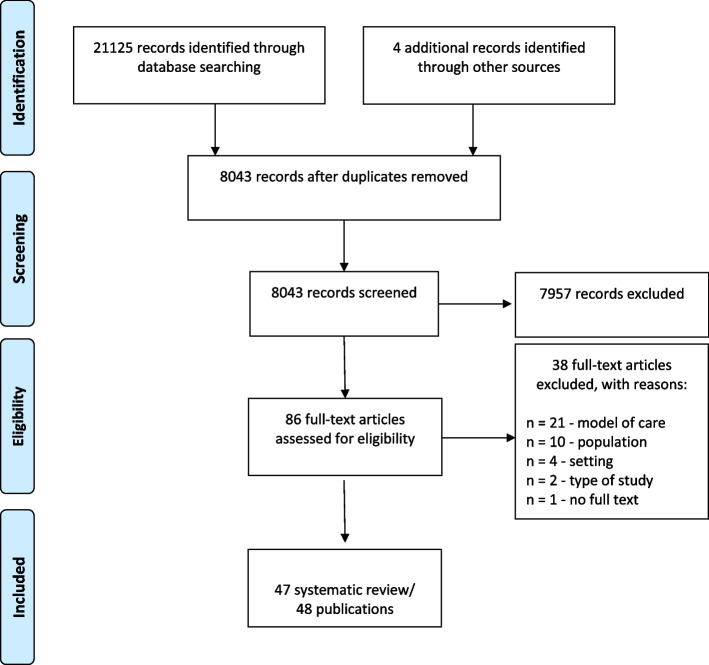
Prisma flow-chart

Our approach was guided by accepted methodological and reporting standards for overviews and mapping reviews [19–21] including PRISMA flow-chart (Fig. 1). We registered the review protocol on PROSPERO (CRD42020166330). We have not made any amendments to the information provided at registration. Our systematic mapping review focused on models of outpatient care.

### Inclusion and exclusion criteria

In the protocol, we decided to include only published systematic reviews. We searched for English- and German-language human studies published since 2000. However, for pragmatic reasons we did not choose a larger period. We only included systematic reviews of models of care for people with chronic heart diseases (coronary heart disease, heart failure, and atrial fibrillation) in outpatient care that met the criteria of the Centre for Reviews and Dissemination (Database of Abstracts of Reviews of Effects, DARE) [22]. Initiation and delivery of interventions (models of care) were required to be linked to outpatient/primary care. Reviews with interventions for specific vulnerable groups (e.g. pregnant woman and palliative care) and infants (0–3 age) were excluded. All other study designs were excluded (Table 1).

**Table 1:** Inclusion and exclusion criteria

	Inclusion criteria	Exclusion criteria
Population	People with chronic heart diseases • Coronary heart disease, • Heart failure and • Atrial fibrillation Age: adults Sex: no restrictions	Specific vulnerable groups (e.g. pregnant woman and palliative care) Age: infants (0–3 age)
Interest	Models of outpatient care	Medical care, e.g. medication, operation, devices
Context	Outpatient care	Inpatient care
Study design	SRs that met at least four of five DARE criteria	Quantitative and qualitative studies
Years considered	January 1, 2000, to June, 30 2021	Publications before January 1, 2000
Language	English- and German-language	Other languages than English or German
Publication status	Published	Preprint

### Data sources and searches

The National Library of Medicine through PubMed, Cochrane Library (Wiley), Web of Science and Scopus were searched for systematic reviews published between January 1, 2000, and June, 30 2021. We used a combination of MeSH and text terms that included terms related to chronic heart diseases, models of care and outpatient care settings. A full search strategy is available in Additional file 1: Search strategy at Pubmed. We used Citavi 6, a reference management software to manage our records and remove duplicates.

### Eligibility screening process

Two groups of reviewers MH, HH and AS, TR—initial search until December 2019; MH, SE and HH, SE—search update, each screening 50% of total) independently screened titles and abstracts for eligibility based on the above selection criteria. All studies reviewed as “yes” or “unsure” by either reviewer team were included in full-text screening. Agreement between both reviewers was required to exclude a study. Where consensus of eligibility was not reached a third reviewer (CA) was consulted. We obtained full texts for all selected systematic reviews, and if study eligibility remained unclear, we discussed again with a third reviewer (CA).

### Data extraction

Two groups of reviewers (MH, CD and HH, SE, each 100% of total) extracted data independently and subsequently reconciled. An Excel spreadsheet for data extraction was developed and piloted by the review team. From each systematic review search dates, objectives, populations, number and types of included studies, number of included participants, components of interventions, and outcomes reported were extracted and summarised.

### Data analysis

We analysed the extracted data from the included SRs with descriptive statistics for reporting frequencies where appropriate. Temporal data were represented in visual graphs to illustrate trends. All other data were presented in tabular form. We inductively categorised MoC based on the titles and summaries of the included SRs. Intervention categories were formed by inductively coding the characteristics of the interventions in the SRs (content analysis) and jointly consented by the review group. We mapped the outcomes onto the taxonomy developed by the COMET initiative [23]. The COMET initiative encourage the development and uptake of core outcome sets: an agreed standard set of outcomes that should be measured and reported, as a minimum, in all clinical trials in specific areas of health or healthcare setting [24]. We categorised by the core areas: death, physiological or clinical, life impact, resource use and adverse events [23]. If an outcome was composite and addressed several core areas, we classified it within each of the relevant domains. All authors reached final consensus on findings, interpretation and text. We did not perform an assessment of the methodological quality of the included SRs.

To perform a comprehensive analysis of the overlap of the included SRs, we used the GROOVE tool and included all primary studies [25]. Besides the calculation of overall Corrected Covered Areas (CCA), GROOVE provides a graphical representation of the overlap among each pair of possible SRs (nodes). A CCA value lower than 5 can be considered as a slight overlap, whereas values greater than or equal to 15 can be considered as a very high overlap (0–5; slight, 6–10; moderate, 11–15; high, and > 15; very high overlap) [26].

## Results

### Studies and study characteristics

After the removing of duplicates, 86 potentially eligible records were identified and screened according to the protocol. We excluded thirty-eight articles after full-text screening, because they did not meet the inclusion criteria; no model of care—21 articles [27–47], no eligible population—ten articles [48–57], no eligible setting—4 articles [58–61], no SR—2 articles [62, 63], no full text available—1 article [64]. Fourty-seven systematic reviews/48 publications, covering 1154 primary studies (including double counting) were included in this review (Fig. 1). Most systematic reviews included models of care for heart failure (42 SRs), in addition to coronary heart disease (4 SRs), and atrial fibrillation (1 SR).

**Fig. 2:**
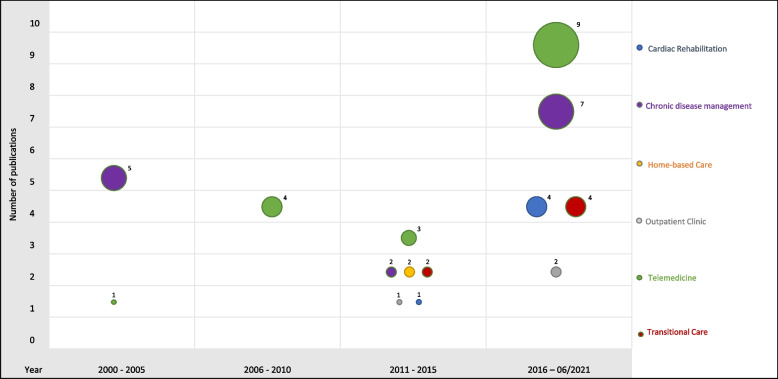
Evidence map — models of care

The number of publications on interventions for patients with chronic heart diseases in outpatient care has increased. While only 21.3% of the systematic reviews included here were published in 2000–2010, 55.3% were published since January 2016 (Fig. 2). The studies included in the SRs were conducted between 1967 and 2021.

The authors of the included SRs performed a systematic search of at least two to a maximum of 16 databases to identify randomised controlled trials, non-randomised controlled trials or other relevant research articles to answer the research question. Twenty-one different databases were used for literature search in the included SRs. The number of included participants ranged from 867 to 28,455. The number of included studies ranged from 6 to 70 studies per systematic review (Additional file 2: Characteristics of the included SRs).

**Fig. 3:**
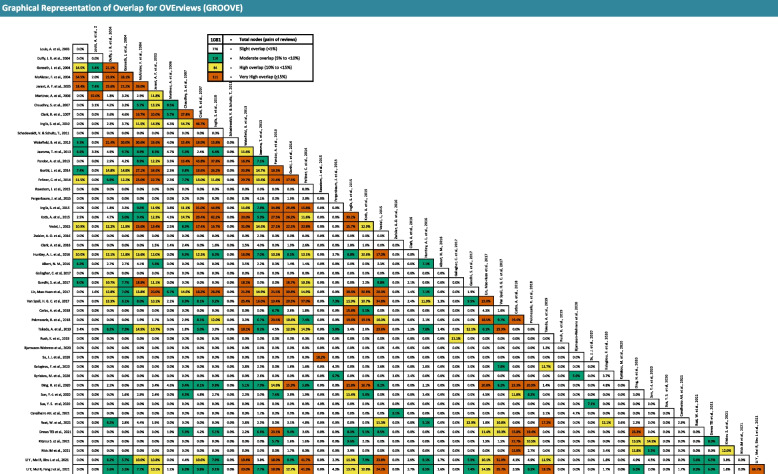
Graphical representation of overlap for overviews (GROOVE)

The CCA in our overlap analysis was 3.08% (slight overlap) for all included systematic reviews. However, we measured a very high overlap (≥ 15%) at 111 of 1081 nodes and a high overlap (10–15%) at further 84 nodes. This means that a large number of studies appeared several times across the systematic reviews (Fig. 3). Five primary studies were included in 15 or more SRs (Table 2).

**Table 2:** Overlap analysis

**Number of columns (number of reviews)**	**c**	**47**
**Number of rows (number of index publications)**	r	499
**Number of included primary studies** **(including double counting)**	N	1206
**Covered area**	N/(rc)	5.14%
**Corrected covered area**	(N-r)/(rc-r)	3.08%
**Interpretation of overlap**	Slight overlap	
**Structural zeros**	X	0
**Corrected covered area** **(adjusting by structural zeros)**	(N-r)/(rc-r-X)	3.08%
**N° of non-overlapped primary studies**	In 1 SR	294
**Number of overlapped primary studies**	In 2 SRs	79
	In 3 SRs	33
	In 4 SRs	14
	In 5 SRs	13
	In 6 SRs	16
	In 7 SRs	10
	In 8 SRs	9
	In 9 SRs	4
	In 10 SRs	8
	In 11 SRs	2
	In 12 SRs	3
	In 13 SRs	1
	In 14 SRs	2
	In 15 or more SRs	5

### Evidence map on models of outpatient care

In our evidence mapping, we identified six models of outpatient care: (a) cardiac rehabilitation, (b) chronic disease management, (c) home-based care, (d) outpatient clinics, (e) telemedicine, and (f) transitional care.

We categorised MoCs interventions according to the following characteristics: remote monitoring of daily biometric data, structured telephone support, video conference, information/education program, symptom monitoring (by staff or patients), regular consultation (e.g. outpatient, hospital), home visits, pharmacologic management, nutrition, coordination, behavioural or psychosocial support, planning for discharge, and exercise, which has been visualised in Additional file 3: Mapping of the single interventions of the included SRs. In addition, we mapped the primary outcomes of the included SRs onto the taxonomy developed by the COMET initiative [23] (Additional file 4: Mapping of the primary outcomes of the included SRs). Outcomes from all core areas were used: in 30 SRs outcomes from the core areas death (65.2%) were included. Physical/clinical outcomes were included in 11 SRs (23.9%), life impact in 25 SRs (54.3%), resource use in 30 SRs (65.2%) and adverse events in 6 SRs (13%).

#### Cardiac rehabilitation

Cardiac rehabilitation (CR) was designed to optimise secondary prevention of chronic heart failure. It has evolved from an exercise-focused program to a comprehensive, multi-component model of care to address risk factors. Indeed, CR societies have published and internationally agreed so-called “core components” of CR, namely, nutritional counselling, risk factor modification, psychosocial management, patient education, and exercise training [65].

We included five SRs with heart failure and CHD indications for the MoC cardiac rehabilitation [66–70]. All included SRs on MoC reported exercise programs. In addition, four out of five included at least one telemonitoring intervention (remote monitoring of daily biometric data, structured telephone support, or video conference). Information/education programs and behavioural or psychosocial support were provided in 60% of the included SRs. One SR each described nutrition and home visits as interventions. Death and life impact are relevant outcomes for cardiac rehabilitation. All systematic reviews used outcomes of these core areas to evaluate interventions. Thus, four SRs employed psychological/clinical outcomes (80%), three SRs resource use outcomes (60%) and one SR adverse events outcomes (20%).

#### Chronic disease management

Definitions of chronic disease management (programs) vary substantially. We therefore included a wider range of approaches, which we considered as “chronic disease management”, e.g. case management [71], chronic care model [72], coordinated care and integrated care [73], and disease management programs [74]. Chronic disease management does not normally involve general coordination of care and preventive services such as flu vaccination [75].

The MoC chronic disease management included only SRs for people with heart failure [39, 76–88]. The included SRs demonstrated three core elements: telemonitoring, coordination and information/education program. Telemonitoring interventions (at least one intervention) were part in 12 SRs (85.7%). Coordination and information/education programs were each included in 71% of SRs (*n* = 10). In addition behavioural or psychosocial support (7; 50%), regular consultation (6; 43%), symptom monitoring (4; 29%), pharmacological management (4; 29%), and home visits (21%) were reported. Outcomes of the core areas death and resource use were reported in seven of the SRs (50%). Life impact outcomes were included in seven SRs (50%), psychological/clinical outcomes in three (21%) and adverse events outcomes in only two systematic reviews (14%).

#### Home-based care

Home-based care aims to improve health and prevent the need for long-term care or immediate care. It includes a variety of home services such as health promotion and teaching, clinical care, end-of-life care, rehabilitation, social adaptation and integration, and support for informal caregivers [89].

The MoC home-based care included only two SRs for people with heart failure [89, 90]. One SR was based only on home visits. The second SR included also information/education, behavioural or psychosocial support, pharmacological management, and coordination interventions. All SRs included outcomes from the core area death. Further outcomes from the core areas life impact and resource use were used in one of the two SRs.

#### Outpatient clinic

Outpatient clinics (often located at hospitals) provide a collaborative, multidisciplinary approach for treatment with cardiologists, nurses, pharmacists, dieticians, psychologists, and social workers. These clinics not only provide optimisation of drug therapy, but also education regarding lifestyle such as diet and exercise, medication compliance and diuretic titration strategies; they serve as a crucial link for patients who develop worsening symptoms and require earlier follow-up or medication adjustment [91].

We included three SRs for the MoC [91–93], one SR each for of the indications heart failure, coronary heart disease and atrial fibrillation. For all indications, information/education and symptom monitoring are listed as interventions. In addition, the following interventions are described for heart failure: regular consultation, pharmacological management, nutrition, coordination, and behavioural or psychosocial support. There were fewer interventions for CHD and AF. In two of the three systematic reviews on outpatient clinics, outcomes from four core areas were used. Outcomes on resource use were included in all three SRs.

#### Telemedicine

Telemedicine (TM) is delivered by all health care professionals using information and communication technologies for the exchange of valid information for diagnosis, treatment and prevention of disease and injuries, research, and evaluation. In addition, TM is used for continuing education of health care providers, all in the interests of advancing the health of individuals and their communities [94].

The majority of SRs included in the MoC telemedicine [95–111] refer to heart failure. One SR investigated telemedicine MoC for people with CHD. All SRs (*n* = 17) offered at least one telemonitoring intervention to their participants: remote monitoring of daily biometric data (16; 94%), structured telephone support (15; 88%) or video conference (2; 12%). In addition, the following interventions were reported in SRs: symptom monitoring (10; 59%), information/education (7; 41%), pharmacological management (5; 29%), behavioural or psychosocial support and home visits (3; 18%), coordination (2; 12%), exercise program and nutrition (1; 6%). Outcomes from four core areas were included in the TM SRs: death (13; 76%), psychological/clinical (2; 6%), life impact (9; 53%), and resource use (12; 71%). The majority of included telemedicine systematic reviews (12; 70.5%) considered outcomes from two core areas (death, ressource use).

#### Transitional care

Transitional care (TC) encompasses a broad range of services and environments designed to promote the safe and timely passage of patients between levels of health care and across care settings [112].

The MoC transitional care included six SRs for people with heart failure [113–118]. Four of the six included SRs reported at least one telemonitoring intervention. In addition, at least 50% of the SRs also described the following interventions: information/education, symptom monitoring, home visits, regular consultation, and coordination. Pharmacological management and behavioural or psychosocial support was only reported in one SR each. Outcomes from the core area death, life impact, and adverse events were included in two of these six SRs each.

## Discussion

We used evidence mapping of systematic reviews to summarise the complex and growing literature on outpatient care for people with chronic heart diseases. We identified six different models of outpatient care (cardiac rehabilitation, chronic disease management, home-based care, outpatient clinics, telemedicine, and transitional care) and described which interventions were used and what outcomes were included to measure their effectiveness. We observed heterogeneity in terms of interventions within as well as commonalities between MoCs. In addition, we identified populations that require further investigation and observed variation in how outcomes were measured. The total overlap between the SRs was calculated as slight. It seems that this effect is due to the diversity of the models of care. Nevertheless, a high and very high overlap was measured at a total of 195 of 1081 nodes, especially within the MoCs on chronic disease management, telemedicine and transitional care.

Due to the large inclusion time frame, we were able to give a broad overview. It is apparent that research and thus the number of publications has increased in the period examined. Fourty-two out of 47 included SRs were on heart failure. This also means that AF and CHD populations are significantly less studied.

Despite the observed heterogeneity in populations, our evidence map shows noteworthy trends. Systematic reviews on chronic disease management [39, 76, 78–88] and telemedicine [95–105, 107–111, 119] have been increasingly published. Telemedicine provides an opportunity to improve outpatient healthcare delivery. Inequalities in access to healthcare and in the provision of healthcare services, caused by difficulties in transportation and, in some cases, lack of healthcare workers, are particularly challenging in rural areas [120]. The evidence map show core elements of models of care combined with additional interventions. Especially, education-related and telemedicine interventions were described more than 50% of the MoCs. The details on how the interventions were implemented are described in varying detail. In addition, the interventions in the primary studies varied in terms of design, for example frequency of sessions, duration, and their providers. This has not been analysed in our study and needs further investigation.

It was remarkable that outcomes were used very heterogeneously in the SRs. Only few SRs measured psychological/clinical and adverse events outcomes. Rather, outcomes of the core areas death, life impacts, and resources are used. Up to the best of our knowledge, there is a lack of a core outcome set for studies on outpatient care of chronic heart disease. Defined and agreed upon outcomes and measurement tools would help to increase comparability of the effectiveness of interventions in primary studies and systematic reviews.

As visual representation, this evidence map summarise the evolving research on outpatient care for people with chronic heart diseases: We only used published systematic reviews for our overview and evidence mapping [18]. As not necessarily required for our objectives, we have not provide an assessment of the methodological quality of the reviews by available instruments such as AMSTAR-2 [121] or ROBIS [122], neither calculate effect sizes in a meta-analysis. We included only systematic reviews that met at least four of five DARE criteria for SR [22]—we relied on the skills of the authors of the included systematic reviews in conducting the literature search, conducting and assessing study quality, selecting and presenting results.

## Conclusion

In an ever-evolving research environment on outpatient care for people with chronic heart diseases, summarising research evidence on models of outpatient care and interventions is becoming increasingly complex, especially for healthcare stakeholders. Our evidence map can help bridge the gap between the available scientific evidence on outpatient care for people with chronic heart diseases and the adoption of this evidence by health systems with an overview of systematic reviews from January 1, 2000, to June 30, 2021, on models of outpatient care.

In addition, evidence maps are also a useful tool to identify gaps in the literature, and to guide future research. Our results show that the comparability of published SRs on MoC is limited due to differences in interventions and outcome measures. For example, physical/clinical outcomes are less considered in SRs. Outpatient care of CHD and AF is poorly studied compared to HF. Thus our evidence map determines the need for primary or secondary research. First of all, it would be helpful to develop a core outcome set to have a consistent set of clinical endpoints for chronic heart disease outpatient care studies. Further, future studies should examine the effects of models of outpatient care or different interventions with adjusted outcome parameters to determine which interventions in the MoCs described here have the most positive effects for the person concerned. This could provide an impetus for practice and policy to develop renewed or new model of outpatient care for patients with chronic heart diseases.

## Supplementary Information

**Additional file 1.** Search strategy at PubMed.

**Additional file 2:** Characteristics of the included SRs.

**Additional file 3: **Mapping of the single interventions of the included SRs.

**Additional file 4: **Mapping of the primary outcomes of the included SRs.

## Data Availability

The data underlying this article are available in the article and in its online supplementary material.
