# Supplementary material for: An evidence map of systematic reviews on models of outpatient care for patients with chronic heart diseases
Source: Syst Rev. 2023 May 6;12:80. doi: 10.1186/s13643-023-02227-z (PMC10163805; doi:10.1186/s13643-023-02227-z)
Supplement: Supplementary file 1 — Additional file 1. Search strategy at PubMed. [file 13643_2023_2227_MOESM1_ESM.docx]

Search strategy at Pubmed

(((((((((((coronary artery disease*[MeSH Terms]) OR coronary heart disease*[MeSH Terms]) OR heart failure[MeSH Terms]) OR cardiovascular disease*[MeSH Terms]) OR atrial fibrillation*[MeSH Terms]) OR auricular fibrillation*[MeSH Terms])) AND ((((((coronary artery disease*) OR coronary heart disease*) OR heart failure) OR cardiovascular disease*) OR atrial fibrillation*) OR auricular fibrillation*))) AND (((((((disease management*[MeSH Terms]) OR care management*[MeSH Terms]) OR delivery of health care*[MeSH Terms]) OR telemedicine[MeSH Terms]) OR rehabilitation*[MeSH Terms])) AND ((((((((((*intervention) OR disease management) OR case management) OR care management) OR delivery of health care) OR telemedicine) OR cardiac rehabilitation*) OR model of care) OR transition) OR system of care))) AND (((((((((mortality) OR major cardiovascular events) OR patient-reported outcome) OR readmission) OR results) OR findings) OR outcome)) AND (((((hospital readmission[MeSH Terms]) OR mortality[MeSH Terms]) OR findings[MeSH Terms]) OR assessment, outcome health care[MeSH Terms]) OR assessment, patient outcome[MeSH Terms]))) AND ((((((((((community health care[MeSH Terms]) OR primary care[MeSH Terms]) OR health, rural[MeSH Terms]) OR urban health[MeSH Terms]) OR general practice*[MeSH Terms]) OR ambulatory care[MeSH Terms]) OR outpatient care[MeSH Terms]) OR *nurse[MeSH Terms])) AND ((((((((community health care) OR primary care) OR rural health) OR urban health) OR general practice*) OR ambulatory care) OR outpatient care) OR nurse)) AND (systematic[sb] AND ("2000/01/01"[PDat] : "2021/06/30"[PDat]) AND (English[lang] OR German[lang]))
