# Supplementary material for: An evidence map of systematic reviews on models of outpatient care for patients with chronic heart diseases
Source: Syst Rev. 2023 May 6;12:80. doi: 10.1186/s13643-023-02227-z (PMC10163805; doi:10.1186/s13643-023-02227-z)
Supplement: Supplementary file 2 — Additional file 2: Characteristics of the included SRs. [file 13643_2023_2227_MOESM2_ESM.docx]

| Included SRs | Study location | Literature published | Source of information | Number of included studies  (eligible studies) | Number of included participants | Main objective |
| --- | --- | --- | --- | --- | --- | --- |
| Albert NM, 2016 | USA | 1990 – 09/ 2015 | 6 databases (PubMed, Google Scholar, MEDLINE, CINAHL, EMBASE, and the Cochrane Library) | 23 (RCT, non-RCT) | 28.455 | “The objective of this review was to evaluate existing transition-of-care models and identify common themes that may minimize exacerbation and rehospitalization, and improve quality of life for patients with heart failure (HF).” |
| Bjarnason-Wehrens B, Nebel R, Jensen K, et al., 2020 | Germany | 1999 - 2019 | 12 databases (PubMed, Cochrane Database of Systematic Reviews, DARE, CENTRAL, CMR, HTA, EED, Web of Science Core Collection, CINAHL, PsychInfo, CCMED, ClinicalTrials.gov) | 25 (RCT) | 4481 | “[…] to evaluate whether or not exercised-based cardiac rehabilitation (ebCR) is effective in reducing all-cause-mortality and hospitalization and improving exercise capacity (peak oxygen uptake (VO2peak)) and QoL in chronic heart failure with left ventricular ejection fraction (HFrEF)-patients with reduced LV-EF 40% on guideline-recommended pharmacoand device-therapy.” |
| Carbo A, Gupta M, Tamariz L, et al., 2018 | USA | 1966 – 05/ 2017 | 4 databases (PubMed, Cochrane Library, CINHAHL, EMBASE) | 11 (10 RCTs and 1 quasi-experimental study) | 3109 | “[…] compare HF patients monitoring with the use of m-Health technologies, to usual care and reported on HF outcomes.” |
| Cavalheiro AH, Silva Cardoso J, Rocha A et al., 2021 | Portugal | Up to 05/ 2020 | 4 databases (MEDLINE, Scopus,  Web of Science, Cochrane Central  Register of Controlled Trials) | 17 (RCT) | 2206 | “Our systematic review focuses on TR effect on HF patients’ management. The aim of the present study is to systematically review the literature, so as to assess the clinical effectiveness of TR in HF outpatient care, when compared with the standard of care in terms of cardiovascular death and heart failure-related hospitalizations. An additional objective comprises the analysis of TR impact on functional capacity, quality of life, cardiovascular safety and cost-effectiveness.” |
| Chaudhry SI, Phillips CO, Stewart SS, et al., 2007 | USA | 1966 – 08/ 2006 | 2 databases (Medline, EMBASE) | 9 (RCT) | 3582 | “[…] provided an in-depth exploration of the details of each intervention, intensity and duration of the programm, the costs of implementing the telemonitoring programm and the effect, the responsibility for acting on the information and the elements of the telemonitorung program.” |
| Clark AM, Wiens KS, Banner D et al., 2016 | Canada | 1995 – 05/ 2014 | 11 data bases (MEDLINE, EMBASE, PsycINFO, CSA Sociological Abstracts, Academic Search Complete, CINAHL, SocINDEX, ISI Web of Science, Cochrane Central Register of Controlled Trials (CENTRAL) and Scopus)  Grey literature was found via Proquest Dissertations and Theses, Google) | 33 (18 RCTs, three mixed methods studies, six pre-test post-test studies and six qualitative studies) | 3355 | “[…] to identify the main mechanisms of heart failure (HF) disease management programs based in hospitals, homes or the community.” |
| Clark RA, Inglis SC, McAlister FA et al., 2007 | Australia | 01/ 2002 – 05/ 2006 | 16 databases (Cochrane Central Register of Controlled Trials, AMED, ISI web of knowledge, HSTAT, Ingenta; Zetoc, LILACS, DARE, Medline, national research register, Web of Science; Cochrane Library, CINHAHL, EMBASE, PsychInfo; web of Science) | 14 (RCT) | 4264 | “Evaluate the effect of remote monitoring strategies in patients with chronic heart failure and wether the effects differed by the type of technology used for the communication of information.” |
| Ding H, Chen SH, Edwards I et al., 2020 | Australia | 01/ 1990 – 02/ 2020 | 4 databases (PubMed, EMBASE, CINAHL, Cochrane Library database) | 26 (RCT) | 11.450 | “[…] to evaluate the effect of different noninvasive telemonitoring strategies. Systematic review and meta-analysis using a novel approach of evaluating the effect of different noninvasive telemonitoring strategies on reduced all-cause mortality and hospitalization.” |
| Drews TEI, Laukkanen J, Nieminen T, 2021 | Finland | 01/ 2004 – 10/ 2020 | 3 databases (Medline, CINAHL, Scopus) | 11 (RCT) | 4291 | “We planned this systematic review and meta-analysis to study an estimate of the effect of non-invasive home telemonitoring (TM) in the treatment of patients with recently decompensated heart failure (HF).” |
| Duffy JR, Hoskins LM, Chen M-C, 2004 | USA | 1998 - 2003 | 2 databases (Medline and CINAHL) | 31 (RCT) | 4653 | “[…] to synthesize available evidence regarding nonpharmacological interventions, that impact hospital readmission and QoL among community-based HF patients.” |
| Feltner C, Jones CD, Cene CW, et al., 2014 | USA | 07/ 2007 – 10/ 2013 | 5 databases (Medline, ClinicalTrials.gov, World Health Organization International Clinical Trials Registry Platform, Cochrane Library, CINAHL) | 47 (RCT) | 8676 | “[…] to determine transitional care interventions for persons with HF for the Effective Health Care Program of the Agency for Healthcare Research and Quality (AHRQ).” |
| Fergenbaum J, Bermingham S, Krahn M et al., 2015 | Canada | 2006 – 01/ 2012 | 5 databases (Medline, Cumulative Index to Nursing and Allied Health Literature, Centre for Reviews and Dissemination, Cochrane Library, EMBASE) | 6 (RCT) | 1400 | “[…] to determine the clinical effectiveness of "Care in the home" (CHM) compared with usual care in patients with chronic heart failure (CHF) and to estimate the cost-effectiveness of CHM compared with usual care in patients with CHF.” |
| Gallagher C, Elliott AD, Wong CX et al., 2017 | Australia | until 02/ 2016 | 3 databases (PubMed, CINHAHL, EMBASE) | 39 (RCT, non-RCT) | 1383 | “[…] to examine the impact of the integrated care approach in the AF population, compared to usual care.” |
| Gandhi S, Mosleh W, Sharma UC et al., 2017 | Canada | 1990 – 01/ 2017 | 3 databases (PubMed, Cochrane Library, EMBASE) | 16 (RCT) | 3999 | “[…] to determine the benefit of a multidisciplinary HFclinic compared with usual care on clinical outcomes.” |
| Gonseth J, Guallar-Castillon P et al., 2004 | Spain | 1966 – 08/ 2003 | 3 databases (Medline, Cochrane Library, EMBASE) | 54 (RCT, non-RCT) | 21.098 | “The aim of this paper was to systematically review the experimental evaluations of DMPs among elderly patients with HF and to ascertain the effectiveness of such programs.” |
| Gorthi J, Hunter CB, Mooss AN et al., 2014 | USA | 01/ 1975 – 08/ 2014 | 3 databases (PubMed (Medline); EBSCOHost SocINDEX, Cochrane Library) | 49 (RCT) | 19.467 | “The purpose of the present systematic review was to critically evaluate all available studies meeting minimal inclusion criteria to define the efficacy of DMPs in reducing hospitalizations and/or mortality in patients with chronic HF.” |
| Huntley AL, Johnson R, King A et al., 2016 | UK | 1985 - 2012 | 4 databases (MEDLINE, EMBASE, CINAHL and PsycINFO), Update: 2015 on MEDLINE | 22 (RCT, non-RCT) | 8626 | “[…] to investigate the effectiveness and related costs of case management (CM) for patients with heart failure (HF) predominantly based in the community in reducing unplanned readmissions and length of stay (LOS).” |
| Inglis SC, Clark RA, Dierckx R et al., 2015 | Australia | 01/ 1966 – 05/ 2006 | 14 databases (Cochrane Central Register of Controlled Trials; AMED; DARE, Medline; SCI-EXPANDED, CPCIS; Proquest Theses and Dissertations, IEEE Xplore, TROVE, Web of Science, Cochrane Library, CINAHL, EMBASE; Health Technology Assessment Database (HTA)) | 41 (RCT) | 13.192 | “[…] to review randomised controlled trials (RCTs) of structured telephone support or telemonitoring compared to standard practice for patients with CHF in order to quantify the effects of these interventions over and above usual care for these patients.” |
| Inglis SC, Clark RA, McAlister FA et al., 2010 | Australia | 01/ 2006 – 11/ 2008 | 8 databases (CENTRAL, DARE, Health Technology Assessment Database (HTA), MEDLINE, EMBASE, CINAHL, AMED,  Science Citation Index Expanded and Conference Citation Index on ISI Web of Knowledge) | 25 (RCT) | 8323 | “[…] to review randomised controlled trials (RCTs) of structured telephone support or telemonitoring compared to standard practice for patients with CHF in order to quantify the effects of these interventions over and above usual care for these patients.” |
| Jaarsma T, Brons M, Kraai I et al., 2013 | Schweden | Until 04/ 2011 | 4 databases (PubMed, Cochrane Library, CINAHL, EMBASE) | 70 (all articles in peer-reviewed journals) | 18.925 | “The objective of this review was to describe which components of HF (home) care are found in research studies addressing homecare interventions in the HF population.” |
| Jerant AF, Nesbitt TS, 2005 | USA | 1966 – 11/ 2004 | 4 databases (Medline, Cochrane Library, CINAHL, PsychINFO) | 33 (RCT) | 13375 | “[…] to summarize and critically review research regarding heart failure disease management (HFDM) programs incorporating telemedicine and to outline critical research gaps and approaches for addressing them.” |
| Kalogirou F, Forsyth F, Kyriakou M et al., 2020 | UK | 2008 - 2018 | 4 databases (CINAHL, Cochrane, Medline, Embase) | 18 (RCT, non-RCT) | 5435 | “The aim of this analysis was to determine if there was evidence that HF DMPs improved outcomes specifically for patients with HfpEF.” |
| Kitsiou S, Vatani H, Paré G et al., 2021 | USA | Until 07/2020 | 4 databases (MEDLINE, CENTRAL, CINAHL, EMBASE) | 16 (RCT) | 4389 | “[…] to evaluate the effects of mobile health (mHealth) interventions compared with usual care in patients with HF.” |
| Kotb A, Cameron C, Hsieh S et al., 2015 | USA | Until 12/ 2012 | 4 databases (Medline, Cochrane Library, CINAHL, EMBASE) | 30 (RCT) | 10.193 | “This analysis will not only examine the potential impact of telemedicine against usual care, it will also examine the comparative effectiveness of these different interventions against one another.” |
| Kyriakou M, Middleton N, Ktisti S et al., 2020 | Cyprus | Up to 03/ 2017 | 3 databases (PubMed, CINAHL, Cochrane Library) | 10 (RCT) | 867 | “[…] to examine the potential effectiveness of supportive care interventions in improving the HRQoL of patients with HF versus a control group.” |
| Li Y, Fu MR, Luo B et al., 2021 | China | 01/ 2009 – 10/ 2019 and  01/ 2014 - 06/ 2020 | 4 databases (MEDLINE, Embase, Chochrane Library, CINAHL) | 38 (RCT) | 10.871 | “[…] to determine the effects of transitional care interventions on all-cause and HF-specific readmissions, emergency department visits and length of hospital stay in patients hospitalized for HF and to gain insights into potential treatment effect modifiers to explain between-study heterogeneity and to explore the relationship between intervention intensity and complexity and treatment effects by dose-response meta-analysis.” |
| Li Y, Fu MR, Fang J et al., 2021 | China | 01/ 2000 - 06/ 2020 | 4 databases (MEDLINE, Embase, Chochrane Library, CINAHL) | 42 (RCT) | 10.784 | “[…] to evaluate the effectiveness and dose-response of transitional care interventions on patient-centered health outcomes of mortality, quality of life, and emotional distress among individuals with heart failure and to identify the trial-level characteristics potentially affecting the overall effectiveness.” |
| Lin M-H, Yuan W-L, Huang T-C et al., 2017 | China | Until 06/ 2016 | 4 databases (Medline, Cochrane Library, CINHAHL, EMBASE) | 39 (RCT) | 11.758 | “[…] to review the literature and provide an update on the effectiveness of telemedicine for treating patient with CHF.” |
| Louis AA, Turner T, Gretton M et al., 2003 | UK | 1966 - 2002 | 3 databases (Medline, Cochrane Library, EMBASE) and Journal of Telemedicine and Telecare | 24 (RCT, non-RCT, observational studies) | 3643 | “[…] to determine the evidence on telemonitoring as a means of reducing hospitalizations in patients with heart failure.” |
| Martinez A, Everss E, Rojo-Alvarez JL et al., 2006 | Spain | 1951 – 05/ 2004 | 10 databases (LILACS, DARE, Medline, NHS center of reviews and dissemination, IEEE Xplore, Current Contents, Telemedicine Information Exchange (TIE), Cochrane Library, INAHTA, CINAHL) and Journal of Telemedicine and Telecare, Telemedicine Journal and e-Health | 42 (RCT, non-RCT, descriptive studies, non-controlled clinical series) | 2303 | “[…] to assess the value of home monitoring for heart failure patients.” |
| McAlister FA, Lawson FM, Teo KK et al., 2001 | Canada | 1966 - 1999 | 6 databases (Medline, Sigle, Cochrane Controlled Trial Registry, the Cochrane Effective Practice and Organization of Care Study Registry, CINAHL, EMBASE) | 11 (RCT) | 2067 | […] to determine whether heart failure disease management programs reduce mortality or rates of hospitalization.” |
| McAlister FA, Stewart S, Ferrua S et al., 2004 | Canada | 1966 - 2003 | 8 databases(Medline: 1966 to 2003, Embase: 1980 to 2003, CINHAHL: 1982 to 2003, Sigle: 1980 to 2003, AMED: 1985 to 2003, Cochrane Controlled Trial Registry, and the Cochrane Effective Practice and Organization of Care Study Registry | 29 (RCT) | 5039 | “[…] to update an earlier systematic review and to investigate which types of programs are most efficacious and to determine whether multidisciplinary strategies improve outcomes for heart failure (HF) patients.” |
| Nick JM, Roberts LR, Petersen AB, 2021 | USA | 1997-2019 | 8 databases (CINAHL, Cochrane Central Register of Controlled Trials, Embase, MEDLINE, Epistemonikos, ProQuest, PsychINFO, Web of Science) | 12 (RCT/NRCT, quasi-experimental) | 1923 | “[…] to examine the effectiveness of telemonitoring versus usual care on self-care behaviors among community-dwelling adults with heart failure.” |
| Pandor A, Thokala P, Gomersall T et al., 2017 | UK | Until 01/ 2012 | 14 databases (including MEDLINE, EMBASE, PsycINFO and The Cochrane Library) and research registers | 21 (randomised controlled trials or observational cohort studies with a contemporaneous control group) | 6395 | “[…] to determine the clinical effectiveness and cost-effectiveness of home telemonitoring (TM) or structured telephone support (STS) strategies compared with usual care.” |
| Pekmezaris R, Tortez L, Williams M et al., 2018 | USA | 01/ 2001 – 11/ 2016 | 6 databases (PubMed; Cochrane Central Register of Controlled Trials; Medline; Web of Science; CINAHL; EMBASE) | 26 (RCT) | 2506 | “[…] to test the effectiveness of home telemonitoring in patients with heart failure for reducing mortality and hospital use.” |
| Raat W, Smeets M, Janssens S et al., 2021 | Belgium | 01/ 2001 to 12/ 2019 | 3 databases (PubMed, Embase, CENTRAL) | 19 (RCT) | 7577 | “[…] to compare the outcomes of different multidisciplinary HF DMPs in relation to their recruitment setting and involvement of primary care health professionals.” |
| Rawstorn JC, Gant N, Direito A et al., 2016 | New Zealand | Until 05/ 2015 | 6 databases (PubMed, Medline, Cochrane Library, CINAHL, EMBASE, PsychINFO) | 11 (RCT) | 1189 | “[…] to determine the benefits of telehealth exCR on exercise capacity and other modifiable cardiovascular risk factors compared with traditional exCR and usual care, among patients with coronary heart disease.” |
| Rush KL., Burton L, Schaab K et al., 2019 | Canada | Unti 10/2018 | 4 databases (PubMed, Medline, CINAHL, EMBASE) | 17 (RCT, quasi-experimental, prospective, retrospective, quantitative description, and qualitative) | 6857 | “The purpose of this study was to provide the first synthesis of evidence for the impact of nurse-led AF clinics on patient, healthcare utilisation and quality of care outcomes.” |
| Schadewaldt V und Schultz T, 2011 | Australia | Until 03/ 2008 | 20 databases for published and 10 databases for unpublished literature | 7 (RCT) | 3246 | “[…] to determine the effectiveness of nurse-led clinics for patients with coronary heart disease.” |
| Son Y-J, Lee Y, Lee H-J, 2020 | Korea | 01/ 2000 – 07/ 2019 | 7 databases (PubMed, PsychInfo, Cochrane, CINAHL, EMBASE, Web of Science, IEEE) | 8 (RCT) | 2534 | “[…] to estimate the effects of mobile phone-based HF intervention, targeted on  improving self-care, on health outcomes.” |
| Su JJ, Yu D, Paguio JT, 2020 | China | 1806 – 04/ 2019 | 9 databases (Medline, EMBASE, CLNAHL, Web of Science, Scopus, PsycINFO, Cochrane Central Register of Controlled Trails, PubMed, CNKI) | 14 (RCT) | 1783 | “[…] to (a) determine the effects of eHealth CR on behavioural, physiological and clinical outcomes and (b) to identify the programme design that may lead to more effective health benefits.” |
| Sua YS, Jiang Y, Thompson DR, 2020 | Singapore | 01/ 2008- 01/ 2019 | 6 databases (PubMed, Cochrane, CINAHL, ProQuest, Scopus, EMBASE, In addition, searches of grey literature such as ongoing trials with outcomes from the Clinical Trials Registry (www.clinicaltrials.gov) and New York Academy of Medicine grey literature report (https://nyam.org/)) | 15 (RCT) | 2978 | “The aim of this study was to synthesize and evaluate the effectiveness of mobile phone-based self-management interventions for medication adherence and change in blood pressure in patients with coronary heart disease.” |
| Takeda A, Martin N, Taylor RS et al., 2019 | UK | 1937 – 01/ 2018 | 5 databases (Cochrane Central Register of Controlled Trials; Medline; CINAHL; EMBASE; DARE) | 47 (RCT) | 10869 | “[…] to compare the effects of different disease management interventions for heart failure (which are not purely educational in focus), with usual care, in terms of death, hospital readmissions, quality of life and cost‐related outcomes.” |
| Van Spall, Harriette G C, Rahman T et al., 2017 | Canada | 2000 - 2015 | 4 databases (PubMed, Embase, CINAHL, and Cochrane Clinical Trials Register) | 53 (RCT) | 12356 | “[…] to compare the effectiveness of transitional care services in decreasing all-cause death and all-cause readmissions following hospitalization for heart failure (HF).” |
| Vedel I und Khanassov V, 2015 | Canada | 1995 – 02/ 2014 | 4 databases (MEDLINE, PsycINFO, EMBASE, and  Cochrane Database of Systematic Reviews) | 41 (RCT) | 11645 | “We aimed to determine the impact of transitional care interventions  (TCIs) on acute health service use by patients with congestive heart failure in primary care and to identify the most effective TCIs and their optimal duration.” |
| Wakefield BJ, Boren SA, Groves PS et al., 2013 | USA | 1995 - 2008 | 3 databases (Cochrane Central Register of Controlled Trials; Medline; CINAHL) | 35 (RCT) | 8071 | “[…] to describe and quantify individual interventions used in multicomponent outpatient heart failure management programs.” |
| Zwisler A-D, Norton RJ, Dean SG et al., 2016 | Denmark | Until 12/ 2015 | 5 databases (Medline; Cochrane Library; CINHAHL; EMBASE; PsychINFO) | 19 (RCT) | 1290 | “[…] to assess the effectiveness of home-based cardiac rehabilitation (CR) for heart failure compared to either usual medical care (i.e. no CR) or center-based CR.” |
