# Supplementary material for: An evidence map of systematic reviews on models of outpatient care for patients with chronic heart diseases
Source: Syst Rev. 2023 May 6;12:80. doi: 10.1186/s13643-023-02227-z (PMC10163805; doi:10.1186/s13643-023-02227-z)
Supplement: Supplementary file 4 — Additional file 4: Mapping of the primary outcomes of the included SRs. [file 13643_2023_2227_MOESM4_ESM.docx]

Mapping of the primary outcomes of the incuded SRs (red-HI, blue – CHD, green - AF)

|  | **Death** | **Physiological/ clinical** | **Life impact** | **Resource use** | **Adverse events** |  |
| --- | --- | --- | --- | --- | --- | --- |
| **Cardiac Rehabilitation** | | | | | |  |
| Bjarnason- W. et al., 2020 |  |  |  |  |  |  |
| Cavalheiro AH et al., 2021 |  |  |  |  |  |  |
| Rawstorn et al., 2016 |  |  |  |  |  |  |
| Su, Yu et al., 2020 |  |  |  |  |  |  |
| Zwisler et al., 2016 |  |  |  |  |  |  |
| **Chronic disease management** | | | | | |  |
| Clark et al., 2016 |  |  |  |  |  |  |
| Duffy et al., 2004 |  |  |  |  |  |  |
| Gallagher et al., 2017 |  |  |  |  |  |  |
| Gonseth et al., 2004 |  |  |  |  |  |  |
| Gorthi et al., 2014 |  |  |  |  |  |  |
| Huntley et al., 2016 |  |  |  |  |  |  |
| Jerant et al., 2005 |  |  |  |  |  |  |
| Kalogirou et al., 2020 |  |  |  |  |  |  |
| Kyriakou et al., 2020 |  |  |  |  |  |  |
| McAlister et al., 2001 |  |  |  |  |  |  |
| McAlister et al., 2004 |  |  |  |  |  |  |
| Raat et al., 2021 |  |  |  |  |  |  |
| Takeda et al., 2019 |  |  |  |  |  |  |
| Wakefield et al., 2013 |  |  |  |  |  |  |
| **Home-based Care** | | | | | |  |
| Fergenbaum et al., 2015 |  |  |  |  |  |  |
| Jaarsma et al., 2013 |  |  |  |  |  |  |
| **Outpatient clinic** |  |  |  |  |  |  |
| Gandhi et al., 2017 |  |  |  |  |  |  |
| Rush et al., 2019 |  |  |  |  |  |  |
| Schadewaldt et al., 2011 |  |  |  |  |  |  |
| **Telemedicine** | | | | | |  |
| Carbo et al., 2018 |  |  |  |  |  |  |
| Chaudhry et al., 2007 |  |  |  |  |  |  |
| Clark et al., 2007 |  |  |  |  |  |  |
| Ding et al., 2020 |  |  |  |  |  |  |
| Drews et al., 2021 |  |  |  |  |  |  |
| Inglis, C et al., 2015 |  |  |  |  |  |  |
| Inglis, C et al., 2010/11 |  |  |  |  |  |  |
| Kitsiou et al., 2021 |  |  |  |  |  |  |
| Kotb et al., 2015 |  |  |  |  |  |  |
| Lin et al., 2017 |  |  |  |  |  |  |
| Louis et al., 2003 |  |  |  |  |  |  |
| Martinez et al., 2006 |  |  |  |  |  |  |
| Nick et al., 2021 |  |  |  |  |  |  |
| Pandor et al., 2013 |  |  |  |  |  |  |
| Pekmezaris et al., 2018 |  |  |  |  |  |  |
| Son et al., 2020 |  |  |  |  |  |  |
| Sua et al., 2020 |  |  |  |  |  |  |
| **Transitional Care** | | | | | |  |
| Albert, N., 2016 |  |  |  |  |  |  |
| Feltner et al., 2014 |  |  |  |  |  |  |
| Li Y et al., 2021a |  |  |  |  |  |  |
| Li Y et al., 2021b |  |  |  |  |  |  |
| Van Spall et al., 2017 |  |  |  |  |  |  |
| Vedel et al., 2015 |  |  |  |  |  |  |
